# A Sparse Recovery Algorithm for Suppressing Multiple Linear Frequency Modulation Interference in the Synthetic Aperture Radar Image Domain

**DOI:** 10.3390/s24103095

**Published:** 2024-05-13

**Authors:** Guanqi Tong, Xingyu Lu, Jianchao Yang, Wenchao Yu, Hong Gu, Weimin Su

**Affiliations:** School of Electronic and Optical Engineering, Nanjing University of Science and Technology, Nanjing 210094, China; tongguanqi@njust.edu.cn (G.T.); yangjianchao999@126.com (J.Y.); wenchao968@163.com (W.Y.); guhong666@njust.edu.cn (H.G.); suweimin@mail.njust.edu.cn (W.S.)

**Keywords:** sparse recovery, radio frequency interference (RFI), synthetic aperture radar (SAR), alternating direction method of multipliers (ADMM)

## Abstract

In synthetic aperture radar (SAR) signal processing, compared with the raw data of level-0, level-1 SAR images are more readily accessible and available in larger quantities. However, an amount of level-1 images are affected by radio frequency interference (RFI), which typically originates from Linear Frequency Modulation (LFM) signals emitted by ground-based radars. Existing research on interference suppression in level-1 data has primarily focused on two methods: transforming SAR images into simulated echo data for interference suppression, or focusing interference in the frequency domain and applying notching filters to reduce interference energy. However, these methods overlook the effective utilization of the interference parameters or are confined to suppressing only one type of LFM interference at a time. In certain SAR images, multiple types of LFM interference manifest bright radiation artifacts that exhibit varying lengths along the range direction while remaining constant in the azimuth direction. It is necessary to suppress multiple LFM interference on SAR images when original echo data are unavailable. This article proposes a joint sparse recovery algorithm for interference suppression in the SAR image domain. In the SAR image domain, two-dimensional LFM interference typically exhibits differences in parameters such as frequency modulation rate and pulse width in the range direction, while maintaining consistency in the azimuth direction. Based on this observation, this article constructs a series of focusing operators for LFM interference in SAR images. These operators enable the sparse representation of dispersed LFM interference. Subsequently, an optimization model is developed that can effectively suppress multi-LFM interference and reduce image loss with the assistance of a regularization term in the image domain. Simulation experiments conducted in various scenarios validate the superior performance of the proposed method.

## 1. Introduction

Synthetic aperture radar (SAR) is an instrumental remote sensing technology renowned for its ability to provide high-resolution imaging capabilities across the globe, regardless of weather conditions. However, SAR imaging quality is susceptible to the adverse effects of diverse and intricate radio frequency interference (RFI). The interferences typically originate from non-intentional sources of electromagnetic devices operating within a similar frequency band, including neighboring satellites in adjacent orbits and ground broadcasting stations, etc. [1,2,3,4,5,6,7]. RFI suppression methods can be divided into two categories: RFI suppression methods based on level-0 products and those based on level-1 products [8].

Parametric, nonparametric, and semi-parametric methods are used for RFI suppression based on level-0 products [9]. The maximum likelihood estimation (MLE) algorithm [10], parameter maximum likelihood (PML) algorithm [11], least mean square (LMS) algorithm [12], and others estimate the parameters of RFI, reconstruct it, and subtract it from the SAR echo data. However, accurate estimation is required for effective RFI suppression. Nonparametric methods, such as the notched filter method, discard the frequency spectrum contaminated by narrowband RFI [13]. The eigen-subspace projection (ESP) method also removes the signal of interest (SOI) that overlaps with RFI in the frequency domain or eigen-subspace. In order to effectively preserve the SOI, semi-parametric methods based on sparse recovery (SR) and low-rank representation (LRR) have been extensively utilized for RFI suppression. In these methods, the sparsity or low-rank characteristics of RFI are incorporated as regularization terms and minimized through an optimization problem [14,15]. The optimization problem also models a regularization term for the SOI to prevent overfitting. Most methods based on SR or LRR focus on accurately capturing the characteristics of SOI through regularization terms. In Ultra-Wideband (UWB) SAR systems based on the pulse, the $L_{1}$ norm of the SOI in the time domain of raw data is utilized as a regularization term to ensure the imaging of strong scattering points [14,16,17,18,19,20]. In pulse-compression-based SAR systems, the $L_{1}$ norm of the distance profile is incorporated as a regularization term in the optimization model [21,22,23]. For semi-parametric methods, the exclusive reliance on the sparsity of the distance profile is inadequate in ensuring the precise recovery of the SOI.

Traditional algorithms mostly utilize level-0 SAR data, specifically in the raw data domain, for interference suppression, without extensively focusing on the characterization of RFI signals. Access to level-0 SAR data is often limited for users, leading them to primarily work with level-1 data [24,25,26,27,28,29]. As a result, it becomes imperative to prioritize the development of interference suppression techniques that are specifically designed for level-1 data.

In SAR systems, the primary source of RFI often stems from ground-based radars. These ground-based radars typically employ the emission of Linear Frequency Modulation (LFM) signals. For level-1 data affected by LFM interference, there are two categories of methods for interference suppression: those based on the simulation of SAR raw data using SAR images and those based on the analysis of SAR images themselves. In the study by Reigber et al. [30], the transformation of synthesized SAR images into a representation where common raw-data interference filtering methods can be applied is discussed. Building upon this, the work of [9] proposes a defocusing procedure to obtain pseudo-echo data and uses an improved ESP method and traditional frequency domain notching filter (FNF) for mitigating different scenarios of RFI. The research published in [31] introduces a robust principal component analysis (RPCA) method to directly remove RFI artifacts caused by LFM signals in single-look complex (SLC) SAR images. The work of [32] proposes a two-dimensional SPECtral ANalysis (2-D SPECAN) for removing LFM radar interference in spaceborne SAR images [33].

For methods based on the simulation of SAR raw data, the defocusing procedure and subsequent RFI suppression involve a complex process. It may lead to errors in polarimetric calibration due to the potential misestimation of calibration parameters in RFI-affected regions. Methods based on the analysis of SAR images adjust thresholds and discard real echoes at the same locations as the interference, resulting in the loss of target scenes. Interference suppression methods based on both categories are typically designed to address LFM interference from a single parameter category. However, in reality, SAR imaging covers a wide area, and there may be multiple ground-based radars emitting LFM interference with multiple parameters. Additionally, considering the long synthetic aperture of SAR, even if there is only one ground-based radar, its operational mode and waveform parameters may switch within the time span of the beam coverage [34,35]. Therefore, it is crucial to research and develop methods that can effectively suppress LFM interference with multiple parameters. These methods should be capable of mitigating the effects of various types of LFM interference to improve the overall quality of SAR imaging.

The work of [15] introduces a RFI suppression model that combines multiple image domain regularizations. RFI suppression is formulated as an optimization problem with regularization terms to characterize both RFI and the SOI. It aims to preserve different types of targets by incorporating various image domain regularizations. The generalized regularization-based RFI suppression model and solver serve as inspiration for the research presented in this paper.

In this paper, by simultaneously focusing on different parameters of interference in the level-1 SAR images, the method effectively suppresses multiple parameters of ground-based LFM interference. Furthermore, with the assistance of a terrain regularization term, it minimizes the loss of terrain information. To begin, the time–frequency characteristics of SAR images in the range and azimuth directions are observed using Short-Time Fourier Transform (STFT) [36,37,38]. It is found that LFM signals are still present in the SAR images, albeit with different parameters. Next, an analysis is conducted to determine the number of LFM interference types contained in SAR images and to identify the parameters for each LFM interference. Matching signals are then constructed in the range and azimuth directions for each LFM interference. Subsequently, a focusing operator is designed for LFM interference in the level-1 image domain, and a joint optimization model is formulated. This model simultaneously optimizes the degree of interference focusing and the loss of terrain information, enabling the recovery of interference signals and terrain images from contaminated images affected by interference.

The rest of this paper is organized as follows. Section 2 introduces the signal model in the echo domain and image domain. Section 3 shows the proposed the algorithm based on ADMM cyclic iteration. Section 4 introduces the experimental results as well as an analysis of different RFI suppression algorithms for point targets and surface targets. Finally, Section 5 concludes this paper.

## 2. Signal Model

### 2.1. Signal Model of LFM Interference in Echo Domain

In a SAR system, it is assumed that the SAR satellite travels along the azimuth direction with a velocity of *v*. Nearby interfering satellites transmit pulse LFM signals with a similar carrier frequency but with different parameters in multiple segments randomly. During the SAR motion, the received SAR signal is contaminated by LFM interference from other satellites. Assuming there are n(n=1…N) types of interference signals, and each type consists of m(m=1…M) pulses, after the carrier frequency removal, the model for the interference signal can be written as

(1) $$\begin{array}{c} s_{\mathrm{rfi}}\left(\eta ;t\right)=\sum_{n=1}^{N}\sum_{m=1}^{M}A_{m,n}\delta_{m,n}\left(\eta \right)\mathrm{rect}\left(\frac{t-\tau_{m,n}}{T_{n}}\right)\\ \;\;\;\;\;\;\;\cdot \exp\left(j\pi k_{n}{\left(t-\tau_{m,n}\right)}^{2}+j2\pi \Delta f_{n}\left(t-\tau_{m,n}\right)\right) \end{array}$$

where η denotes slow time in the azimuth dimension and *t* donates fast time in the range dimension. $A_{m,n}$ and $\tau_{m,n}$ denote the amplitude and time delay of the *m*-th pulse of the *n*-th kind of LFM interference, respectively. $\tau_{m,n}$ is calculated by dividing the slant range between the source of interference and the SAR antenna phase center by the speed of light. $T_{n}$ and $k_{n}$ are the pulse width and frequency modulation rate, respectively. $\Delta f_{n}$ is the carrier frequency difference between the *n*-th kind of LFM interference signal and the SAR signal. The RFI exhibits random and short-duration characteristics. Considering that SAR operates in a step-by-step manner, in the azimuth direction, the interference signal can be represented as an impulse function. Therefore, it is represented using the Dirac function $\delta_{m,n}$.

The echo received after illuminating the targets with the transmitted signal from the SAR satellite can be written as

(2) $$\begin{array}{c} s_{0}\left(\eta ;t\right)=\sum_{i=1}^{K}A_{i}a_{a}\left(\eta \right)\mathrm{rect}\left(\frac{t-2R_{i}/c}{T_{0}}\right)\\ \;\;\;\;\;\cdot \exp\left(j\pi k_{0}{\left(t-\frac{2R_{i}}{c}\right)}^{2}\right)\exp\left(-j\frac{4\pi }{\lambda }R_{i}\right) \end{array}$$

where $A_{i}\left(i=1\ldots K\right)$ denotes the radar cross section (RCS) of each target. $a_{a}$ donates the azimuth window function. $T_{0}$ and $k_{0}$ donate the pulse width and frequency modulation rate of the transmitted signal, respectively. λ denotes the wavelength of the carrier signal. $R_{i}$ donates the slant range between the *i*-th target and the instantaneous position of the radar platform.

The model of the total signal can be written as

(3) $$s\left(\eta ;t\right)=s_{0}\left(\eta ;t\right)+s_{\mathrm{rfi}}\left(\eta ;t\right)+s_{z}$$

where $s_{z}$ denotes the Gaussian white noise.

### 2.2. Signal Model of LFM Interference in Image Domain

The SAR received signal, after carrier removal and pulse compression processing, forms an L1 level Single-Look Complex (SLC) image. The reference signal for matched filtering can be written as

(4) $$s_{\mathrm{ref}}\left(\eta ;t\right)=\mathrm{rect}\left(\frac{t}{T_{0}}\right)\exp\left(-j\pi k_{0}t^{2}\right)a_{a}\left(\eta \right)\exp\left(-j\pi k_{a}\eta^{2}\right)$$

where $k_{a}$ denotes the frequency modulation rate in the azimuth dimension. After matched filtering, a target is represented by a sinc-like shape. The interference in the azimuth direction is modulated from a Dirac function to the LFM signal. The LFM signal has a frequency modulation opposite to the azimuth signal of $s_{0}\left(\eta ;t\right)$. In the range direction, the interference and the reference signal of matched filtering do not match, resulting in changes in the frequency modulation rate and pulse width of the LFM interference. The LFM interference in the image domain can be written as

(5) $$\begin{array}{cc} s_{\mathrm{rfi}}^{\prime }\left(\eta^{\prime };t^{\prime }\right)= & \sum_{n=1}^{N}\sum_{m=1}^{M}A_{m,n}\mathrm{rect}\left(\frac{\eta^{\prime }-\eta_{m,n}}{T_{a}}\right)\exp\left(j\pi k_{a}^{\prime }{\left(\eta^{\prime }-\eta_{m,n}\right)}^{2}\right)\\ & \cdot \mathrm{rect}\left(\frac{t^{\prime }-\tau_{m,n}}{T_{n}^{\prime }}\right)\exp\left(j\pi k_{n}^{\prime }{\left(t^{\prime }-\tau_{m,n}\right)}^{2}+j2\pi \Delta f_{n}\left(t^{\prime }-\tau_{m,n}\right)\right) \end{array}$$

where $\eta^{\prime }$ and $t^{\prime }$ are the azimuth time and range time in the image domain. $T_{a}$ denotes the synthetic aperture time, while $k_{a}^{\prime }$ is opposite to the azimuth frequency modulation rate of the SAR system. According to the work in [32], $k_{n}\neq k_{0},\;k_{n}^{\prime }=-\left(k_{n}k_{0}\right)/\left(k_{n}-k_{0}\right)$ and $T_{n}^{\prime }=T_{0}\left|k_{n}-k_{0}\right|/k_{0}$.

Casting the above model in matrix form allows the problem to be analyzed more clearly. Assuming there are two types of interference, denoted as $R_{1}$ and $R_{2}$, the model for the SAR received signal in Formula (3) can be written as

(6) $$S=S_{0}+R_{1}+R_{2}+Z$$

(7) $$S_{0}=G_{0}\left(Y_{0}\right)$$

where $Y_{0}$ represents the location of targets. Any non-zero element at a certain position in the matrix $Y_{0}$ indicates the presence of a target at that location. $G_{0}\left(\cdot \right)$ denotes the transmission operator of the signal. The process of matched filtering can be represented by the operator $G_{0}^{-1}\left(\cdot \right)$, and their specific forms can be written as

(8) $$\begin{array}{c} G_{0}\left(\cdot \right)=F_{a}^{-1}\left[F_{r}^{-1}\left\{F_{a}\left[F_{r}\left(\cdot \right)⊙\Theta_{\mathrm{rc}}\right]⊙\Theta_{\mathrm{rcmc}}\right\}⊙\Theta_{\mathrm{ac}}\right]\\ G_{0}^{-1}\left(\cdot \right)=F_{r}^{-1}\left[F_{a}^{-1}\left\{F_{r}\left[F_{a}\left(\cdot \right)⊘\Theta_{\mathrm{ac}}\right]⊘\Theta_{\mathrm{rcmc}}\right\}⊘\Theta_{\mathrm{rc}}\right] \end{array}$$

where the operators $F_{a}\left(\cdot \right)$ and $F_{r}\left(\cdot \right)$ represent the Fourier transforms in the azimuth and range dimensions, respectively. The superscript ^−1^ denotes the inverse transform. The range compression, azimuth compression, and bulk range cell migration correction (RCMC) operators are denoted as $\Theta_{\mathrm{rc}}$, $\Theta_{\mathrm{ac}}$, and $\Theta_{\mathrm{rcmc}}$, respectively. The Hadamard product and division operators are represented by ⊙ and ⊘, respectively.

The received signal in Formula (6) is processed through matched filtering to obtain the level-1 SAR image, written as

(9) $$\begin{array}{cc} Y & =G_{0}^{-1}\left(G_{0}\left(Y_{0}\right)+R_{1}+R_{2}+Z\right)\\ & =Y_{0}+G_{0}^{-1}\left(R_{1}\right)+G_{0}^{-1}\left(R_{2}\right)+G_{0}^{-1}\left(Z\right) \end{array}$$

Due to the effects of sidelobes, noise, and interference, Y is only an approximation of $Y_{0}$. Similarly, $G_{i}^{-1}\left(\cdot \right)\left(i=1,2\right)$ denotes the focusing process on the interference signal $R_{i}$. $Y_{i}$ denotes the result of focusing on the interference of the SAR image Y. Taking i=1 as an example, it yields the interference signal focused in the image domain, written as

(10) $$Y_{1}\approx G_{1}^{-1}\left(Y\right)\approx G_{1}^{-1}\left(G_{0}^{-1}\left(R_{1}\right)\right)$$

Under the assumption of sparsity in SAR images, when interference is focused in the image domain, its distribution also satisfies the sparsity condition. Therefore, the optimization problem of interference suppression can be written as

(11) $$\begin{array}{c} \min_{Y_{0},Y_{1},Y_{2}}\lambda_{0}∥Y_{0}{∥}_{1}+\lambda_{1}∥Y_{1}{∥}_{1}+\lambda_{2}{∥Y_{2}∥}_{1},\\ s.t.\;\;∥Y-Y_{0}-G_{1}\left(Y_{1}\right)-G_{2}\left(Y_{2}\right){∥}_{F}^{2}<\epsilon \end{array}$$

where $\lambda_{i}\left(i=0,1,2\right)$ is the scalar parameters of $Y_{i}$ regularizations. $L_{1}$ norm ‘${∥\cdot ∥}_{1}$’ minimizes the sparsity of regular terms. The model’s objective is to minimize the sparsity of the images subject to the constraint that the Frobenius norm ‘${∥\cdot ∥}_{F}$’ of the difference between the original image and the reconstructed image is less than ϵ. The objective of this optimization problem is to suppress the impact of interference by minimizing the sum of sparse terms. The solution to the optimization problem will separate the focused interference ‘image’ from the reconstructed image with a lessened interference impact, thereby achieving interference suppression.

## 3. Sparse Recovery Algorithm Based on ADMM Optimization

This paper presents an algorithm for suppressing LFM interference in SAR images named LFMIS-JointSR. The algorithm utilizes an ADMM-based iterative approach to solve the joint sparse optimization problem, aiming to achieve more accurate estimation results for SAR images and focused images of different interference. The fundamental concept behind ADMM is to reframe the original problem into a sequence of subproblems by introducing Lagrange multipliers. In the case of the joint sparse optimization problem depicted by Formula (11), the Lagrangian function L is denoted as

(12) $$\begin{array}{c} L(Y,Y_{0},Y_{1},Y_{2},U_{0},U_{1},U_{2},\Phi_{0},\Phi_{1},\Phi_{2})=\frac{1}{2}{∥Y-Y_{0}-G_{1}\left(Y_{1}\right)-G_{2}\left(Y_{2}\right)∥}_{F}^{2}\\ \;\;\;\;\;+\lambda_{0}{∥U_{0}∥}_{1}+\lambda_{1}{∥U_{1}∥}_{1}+\lambda_{2}{∥U_{2}∥}_{1}\\ \;\;\;\;\;+\langle \Phi_{0},U_{0}-Y_{0}\rangle +\langle \Phi_{1},U_{1}-Y_{1}\rangle +\langle \Phi_{2},U_{2}-Y_{2}\rangle \\ \;\;\;\;\;+\frac{\mu }{2}{∥U_{0}-Y_{0}∥}_{F}^{2}+\frac{\mu }{2}{∥U_{1}-Y_{1}∥}_{F}^{2}+\frac{\mu }{2}{∥U_{2}-Y_{2}∥}_{F}^{2} \end{array}$$

where $U_{i}\left(i=0,1,2\right)$ denote the augmented variables. μ>0 is a penalty parameter. $\Phi_{i}=\mu \left(U_{i}-Y_{i}\right)$ denote the Lagrangian multipliers. By rewriting $U_{i}-Y_{i}$ in the penalty term ${∥U_{i}-Y_{i}∥}_{F}^{2}$ as $U_{i}-Y_{i}+\frac{\Phi_{i}}{\mu }-\frac{\Phi_{i}}{\mu }$, and then splitting it, combining the inner product terms, and dividing the Lagrangian function L by μ, we can obtain the new Lagrangian function, denoted as

(13) $$\begin{array}{c} L(Y,Y_{0},Y_{1},Y_{2},U_{0},U_{1},U_{2},\Phi_{0},\Phi_{1},\Phi_{2})=\frac{1}{2}{∥X-G_{1}\left(Y_{1}\right)-G_{2}\left(Y_{2}\right)-Y_{0}∥}_{F}^{2}\\ \;\;\;\;\;\;\;+\lambda_{0}{∥U_{0}∥}_{1}+\lambda_{1}{∥U_{1}∥}_{1}+\lambda_{2}{∥U_{2}∥}_{1}\\ \;\;\;\;\;\;\;+\frac{1}{2}\left({∥U_{0}-Y_{0}+\frac{\Phi_{0}}{\mu }∥}_{F}^{2}-{∥\frac{\Phi_{0}}{\mu }∥}_{F}^{2}\right)\\ \;\;\;\;\;\;\;+\frac{1}{2}\left({∥U_{1}-Y_{1}+\frac{\Phi_{1}}{\mu }∥}_{F}^{2}-{∥\frac{\Phi_{1}}{\mu }∥}_{F}^{2}\right)\\ \;\;\;\;\;\;\;+\frac{1}{2}\left({∥U_{2}-Y_{2}+\frac{\Phi_{2}}{\mu }∥}_{F}^{2}-{∥\frac{\Phi_{2}}{\mu }∥}_{F}^{2}\right) \end{array}$$

Next, the solution to the subproblems will be presented through the following steps of updating variables.

1. Optimize the augmented variables $U_{i}$:
  By holding the other variables constant and assuming that they have been pre-estimated, the optimization problem derived from the Lagrangian function, as indicated in Formula (13), can be simplified as
  (14) $$\begin{array}{c} U_{0}^{\left(k+1\right)}=\arg\min_{U_{0}}\frac{\lambda_{0}}{\mu^{\left(k\right)}}∥U_{0}^{\left(k\right)}{∥}_{1}+\frac{1}{2}∥U_{0}^{\left(k\right)}-Y_{0}^{\left(k\right)}+\frac{\Phi_{0}^{\left(k\right)}}{\mu^{\left(k\right)}}{∥}_{F}^{2}\\ U_{1}^{\left(k+1\right)}=\arg\min_{U_{1}}\frac{\lambda_{1}}{\mu^{\left(k\right)}}∥U_{1}^{\left(k\right)}{∥}_{1}+\frac{1}{2}∥U_{1}^{\left(k\right)}-Y_{1}^{\left(k\right)}+\frac{\Phi_{1}^{\left(k\right)}}{\mu^{\left(k\right)}}{∥}_{F}^{2}\\ U_{2}^{\left(k+1\right)}=\arg\min_{U_{2}}\frac{\lambda_{2}}{\mu^{\left(k\right)}}∥U_{2}^{\left(k\right)}{∥}_{1}+\frac{1}{2}∥U_{2}^{\left(k\right)}-Y_{2}^{\left(k\right)}+\frac{\Phi_{2}^{\left(k\right)}}{\mu^{\left(k\right)}}{∥}_{F}^{2} \end{array}$$
  where the superscript *k* represents the iteration counter for the variables. By utilizing the soft threshold algorithm to solve the aforementioned optimization problems, we can obtain
  (15) $$\begin{array}{c} U_{0}^{\left(k+1\right)}=ST\left(Y_{0}^{\left(k\right)}-\frac{\Phi_{0}^{\left(k\right)}}{\mu^{\left(k\right)}},\frac{\lambda_{0}}{\mu^{\left(k\right)}}\right)\\ U_{1}^{\left(k+1\right)}=ST\left(Y_{1}^{\left(k\right)}-\frac{\Phi_{1}^{\left(k\right)}}{\mu^{\left(k\right)}},\frac{\lambda_{1}}{\mu^{\left(k\right)}}\right)\\ U_{2}^{\left(k+1\right)}=ST\left(Y_{2}^{\left(k\right)}-\frac{\Phi_{2}^{\left(k\right)}}{\mu^{\left(k\right)}},\frac{\lambda_{2}}{\mu^{\left(k\right)}}\right) \end{array}$$
  where
  (16) $$ST{\left(A,b\right)}_{i,j}=\left\{\begin{array}{cc} A_{i,j}, & \mathrm{if}\;\left|A_{i,j}\right|>b\\ 0, & \mathrm{otherwise} \end{array}\right.$$
2. Optimize $Y_{i}$:
  Similarly, by holding the other variables, including the previously estimated $U_{i}$, constant, the optimization problems for $Y_{i}$ can be written as
  (17) $$\begin{array}{c} Y_{0}^{\left(k+1\right)}=\arg\min_{Y_{0}}\frac{1}{2\mu^{\left(k\right)}}∥Y-Y_{0}^{\left(k\right)}-G_{1}\left(Y_{1}^{\left(k\right)}\right)-G_{2}\left(Y_{2}^{\left(k\right)}\right){∥}_{F}^{2}\\ \;\;\;\;\;\;\;+\frac{1}{2}∥U_{0}^{\left(k+1\right)}-Y_{0}^{\left(k\right)}+\frac{\Phi_{0}^{\left(k\right)}}{\mu^{\left(k\right)}}{∥}_{F}^{2}\\ Y_{1}^{\left(k+1\right)}=\arg\min_{Y_{1}}\frac{1}{2\mu^{\left(k\right)}}∥Y-Y_{0}^{\left(k+1\right)}-G_{1}\left(Y_{1}^{\left(k\right)}\right)-G_{2}\left(Y_{2}^{\left(k\right)}\right){∥}_{F}^{2}\\ \;\;\;\;\;\;\;+\frac{1}{2}∥U_{1}^{\left(k+1\right)}-Y_{1}^{\left(k\right)}+\frac{\Phi_{1}^{\left(k\right)}}{\mu^{\left(k\right)}}{∥}_{F}^{2}\\ Y_{2}^{\left(k+1\right)}=\arg\min_{Y_{2}}\frac{1}{2\mu^{\left(k\right)}}∥Y-Y_{0}^{\left(k+1\right)}-G_{1}\left(Y_{1}^{\left(k+1\right)}\right)-G_{2}\left(Y_{2}^{\left(k\right)}\right){∥}_{F}^{2}\\ \;\;\;\;\;\;\;+\frac{1}{2}∥U_{2}^{\left(k+1\right)}-Y_{2}^{\left(k\right)}+\frac{\Phi_{2}^{\left(k\right)}}{\mu^{\left(k\right)}}{∥}_{F}^{2} \end{array}$$
  Based on the derivation in [39], it is demonstrated that the application of an operator on a matrix leaves its Frobenius norm unaffected. Setting the first-order derivative to zero yields the minimizers of $Y_{i}$. The results are as follows:
  (18) $$\begin{array}{c} Y_{0}^{\left(k+1\right)}=\frac{1}{\mu^{\left(k\right)}+1}\left[\mu^{\left(k\right)}U_{0}^{\left(k+1\right)}+\Phi_{0}^{\left(k\right)}+Y-G_{1}\left(Y_{1}^{\left(k\right)}\right)-G_{2}\left(Y_{2}^{\left(k\right)}\right)\right]\\ Y_{1}^{\left(k+1\right)}=\frac{1}{\mu^{\left(k\right)}+1}\left[\mu^{\left(k\right)}U_{1}^{\left(k+1\right)}+\Phi_{1}^{\left(k\right)}+G_{1}^{-1}\left(Y-Y_{0}^{\left(k+1\right)}-G_{2}\left(Y_{2}^{\left(k\right)}\right)\right)\right]\\ Y_{2}^{\left(k+1\right)}=\frac{1}{\mu^{\left(k\right)}+1}\left[\mu^{\left(k\right)}U_{2}^{\left(k+1\right)}+\Phi_{2}^{\left(k\right)}+G_{2}^{-1}\left(Y-Y_{0}^{\left(k+1\right)}-G_{1}\left(Y_{1}^{\left(k+1\right)}\right)\right)\right] \end{array}$$
3. Update the Lagrange multipliers $\Phi_{i}$ and penalty parameter μ:
  Respectively, the Lagrangian multipliers and the penalty parameter are updated via
  (19) $$\begin{array}{c} \Phi_{0}^{\left(k+1\right)}=\Phi_{0}^{\left(k\right)}+\mu^{\left(k\right)}\left(U_{0}^{\left(k+1\right)}-Y_{0}^{\left(k+1\right)}\right)\\ \Phi_{1}^{\left(k+1\right)}=\Phi_{1}^{\left(k\right)}+\mu^{\left(k\right)}\left(U_{1}^{\left(k+1\right)}-Y_{1}^{\left(k+1\right)}\right)\\ \Phi_{2}^{\left(k+1\right)}=\Phi_{2}^{\left(k\right)}+\mu^{\left(k\right)}\left(U_{2}^{\left(k+1\right)}-Y_{2}^{\left(k+1\right)}\right) \end{array}$$
  and
  (20) $$\mu^{\left(k+1\right)}=\min\left(\mu_{\max},\rho \mu^{\left(k\right)}\right)$$
  where the constant ρ>1 is employed to facilitate the acceleration of convergence. $\mu_{\max}$ denotes the maximum permissible value of μ.
4. Termination criteria for iterations:
  The termination criteria for iterations include the following:
  - The maximum number of iterations, denoted as $K_{\max}$, is reached;
  - The objective function value between consecutive iterations falls below the predefined threshold, i.e., $∥Y-Y_{0}-G_{1}\left(Y_{1}\right)-G_{2}\left(Y_{2}\right){∥}_{F}^{2}/{∥Y∥}_{F}^{2}<\epsilon$.

The proposed ADMM algorithm can be summarized as follows:

(21) $$\begin{array}{c} U_{0}^{\left(k+1\right)}=ST\left(Y_{0}^{\left(k\right)}-\frac{\Phi_{0}^{\left(k\right)}}{\mu^{\left(k\right)}},\frac{\lambda_{0}}{\mu^{\left(k\right)}}\right) \end{array}$$

(22) $$\begin{array}{c} U_{1}^{\left(k+1\right)}=ST\left(Y_{1}^{\left(k\right)}-\frac{\Phi_{1}^{\left(k\right)}}{\mu^{\left(k\right)}},\frac{\lambda_{1}}{\mu^{\left(k\right)}}\right) \end{array}$$

(23) $$\begin{array}{c} U_{2}^{\left(k+1\right)}=ST\left(Y_{2}^{\left(k\right)}-\frac{\Phi_{2}^{\left(k\right)}}{\mu^{\left(k\right)}},\frac{\lambda_{2}}{\mu^{\left(k\right)}}\right) \end{array}$$

(24) $$\begin{array}{c} Y_{0}^{\left(k+1\right)}=\frac{1}{\mu^{\left(k\right)}+1}\left[\mu^{\left(k\right)}U_{0}^{\left(k+1\right)}+\Phi_{0}^{\left(k\right)}+Y-G_{1}\left(Y_{1}^{\left(k\right)}\right)-G_{2}\left(Y_{2}^{\left(k\right)}\right)\right] \end{array}$$

(25) $$\begin{array}{c} Y_{1}^{\left(k+1\right)}=\frac{1}{\mu^{\left(k\right)}+1}\left[\mu^{\left(k\right)}U_{1}^{\left(k+1\right)}+\Phi_{1}^{\left(k\right)}+G_{1}^{-1}\left(Y-Y_{0}^{\left(k+1\right)}-G_{2}\left(Y_{2}^{\left(k\right)}\right)\right)\right] \end{array}$$

(26) $$\begin{array}{c} Y_{2}^{\left(k+1\right)}=\frac{1}{\mu^{\left(k\right)}+1}\left[\mu^{\left(k\right)}U_{2}^{\left(k+1\right)}+\Phi_{2}^{\left(k\right)}+G_{2}^{-1}\left(Y-Y_{0}^{\left(k+1\right)}-G_{1}\left(Y_{1}^{\left(k+1\right)}\right)\right)\right] \end{array}$$

(27) $$\begin{array}{c} \Phi_{0}^{\left(k+1\right)}=\Phi_{0}^{\left(k\right)}+\mu^{\left(k\right)}\left(U_{0}^{\left(k+1\right)}-Y_{0}^{\left(k+1\right)}\right) \end{array}$$

(28) $$\begin{array}{c} \Phi_{1}^{\left(k+1\right)}=\Phi_{1}^{\left(k\right)}+\mu^{\left(k\right)}\left(U_{1}^{\left(k+1\right)}-Y_{1}^{\left(k+1\right)}\right) \end{array}$$

(29) $$\begin{array}{c} \Phi_{2}^{\left(k+1\right)}=\Phi_{2}^{\left(k\right)}+\mu^{\left(k\right)}\left(U_{2}^{\left(k+1\right)}-Y_{2}^{\left(k+1\right)}\right) \end{array}$$

(30) $$\begin{array}{c} \mu^{\left(k+1\right)}=\min\left(\mu_{\max},\rho \mu^{\left(k\right)}\right) \end{array}$$

The steps of the proposed algorithm are depicted in Algorithm 1.   
**Algorithm 1:** Multiple LFM Interference Suppression Algorithm Based on Joint Sparse Regularization  **Input:** Complex-valued SAR image Y  **Output:** Recovered complex-valued SAR image $Y_{0}$, LFM interference image $Y_{1}$ and $Y_{2}$1  Initialize k=0;2  Initialize $U_{0_{\left(0\right)}},U_{1_{\left(0\right)}},U_{2_{\left(0\right)}},Y_{0_{\left(Y\right)}},Y_{1_{\left(G_{1}^{-1}\left(Y\right)\right)}},Y_{2_{\left(G_{2}^{-1}\left(Y\right)\right)}},\Phi_{0_{\left(0\right)}},\Phi_{1_{\left(0\right)}},\Phi_{2_{\left(0\right)}},\mu_{\left(\mu_{0}\right)}$;3  **Repeat**4      k=k+1;5      calculate $U_{0}^{\left(k+1\right)},U_{1}^{\left(k+1\right)},U_{2}^{\left(k+1\right)}$ by (21)–(23);6      calculate $Y_{0}^{\left(k+1\right)},Y_{1}^{\left(k+1\right)},Y_{2}^{\left(k+1\right)}$ by (24)–(26);7      calculate $\Phi_{0}^{\left(k+1\right)},\Phi_{1}^{\left(k+1\right)},\Phi_{2}^{\left(k+1\right)}$ by (27)–(29);8      calculate μ by (30);9  **Until** $k\geq K_{\max}$ or $∥Y-Y_{0}-G_{1}\left(Y_{1}\right)-G_{2}\left(Y_{2}\right){∥}_{F}^{2}/{∥Y∥}_{F}^{2}<\epsilon$

## 4. Experiment and Analysis

In this section, the effectiveness of the proposed method will be validated using simulated SAR data. The simulation parameters are provided in Table 1. Several methods, namely FNF, PCA, RPCA, and LFMIS-JointSR, were utilized for conducting interference suppression in the simulation experiments. The objective was to compare their respective outcomes based on the target-to-background ratio (TBR) and mean square error (MSE). In Section 4.1, comparisons are conducted between various interference conditions and target scenarios, as shown in Table 2. The effectiveness of the proposed algorithms was demonstrated through the use of the TBR metric. In Section 4.2, the performance of different algorithms under different Signal-to-Interference Ratio (SIR) conditions was compared using the MSE metric, showcasing the robustness of the proposed algorithm. In Section 4.3, a quantitative comparison was carried out to better evaluate the proposed method. This comparison included a recently published similar work [32] as well as traditional algorithms.

In addition, it is worth noting that the signal and interference were processed in different domains. When LFM interference is subjected to matched filtering, the resulting signal exhibits a high sidelobe level when processed through a soft thresholding algorithm followed by an inverse-matched filtering transformation. Therefore, for the imaging operator of LFM interference, a Chirp Scaling (CS) imaging operator was experimentally adopted. Considering ${\mathrm{RFI}}_{1}$ as an example, the imaging operator can be written as

(31) $$\begin{array}{c} G_{1}\left(\cdot \right)=F_{a}^{-1}\left[F_{r}^{-1}\left(F_{r}\left[F_{a}\left(\cdot \right)⊙\Theta_{\mathrm{rcmc}}^{\prime }\right]⊙\Theta_{\mathrm{rc}}^{\prime }\right)⊙\Theta_{\mathrm{ac}}^{\prime }⊙\Theta_{\mathrm{pc}}^{\prime }\right] \end{array}$$

(32) $$\begin{array}{c} G_{1}{\left(\cdot \right)}^{-1}=F_{a}^{-1}\left[F_{r}^{-1}\left(F_{r}\left[F_{a}\left(\cdot \right)⊘\Theta_{\mathrm{pc}}^{\prime }\right]⊘\Theta_{\mathrm{ac}}^{\prime }\right)⊘\Theta_{\mathrm{rc}}^{\prime }⊙\Theta_{\mathrm{rcmc}}^{\prime }\right] \end{array}$$

where $\Theta_{\mathrm{rcmc}}^{\prime }$ and $\Theta_{\mathrm{pc}}^{\prime }$ denote the RCMC and additional phase correction operators of the Chirp Scaling algorithm, respectively. $\Theta_{\mathrm{rc}}^{\prime }$ and $\Theta_{\mathrm{ac}}^{\prime }$ denote the range and azimuth compression operators, respectively.

MSE can be used to assess the discrepancy between the image obtained through the RFI suppression method and the original RFI-free image. It is defined as

(33) $$\mathrm{MSE}=10\log_{10}\frac{∥Y-\hat{Y}{∥}_{F}^{2}}{{∥Y∥}_{F}^{2}}$$

where Y denotes the original RFI-free image. $\hat{Y}$ denotes the recovered image after interference suppression. A smaller MSE serves as an indicator of superior performance in interference suppression.

TBR is commonly used to assess imaging quality [40]. It is defined as

(34) $$\mathrm{TBR}=10\log_{10}\frac{\sum ∥\hat{Y}{\left(T\right)∥}_{F}^{2}}{\frac{1}{N_{B}}\sum {∥\hat{Y}\left(B\right)∥}_{F}^{2}}$$

Respectively, T and B are the image patch containing a strong target and background, whose pixel values in the recovered image $\hat{Y}$ are denoted as $\hat{Y}\left(T\right)$ and $\hat{Y}\left(B\right)$. $N_{B}$ denotes the number of pixels in B. A higher TBR signifies enhanced performance in effectively recovering strong targets while minimizing the average sidelobe level.

**Figure 1:**
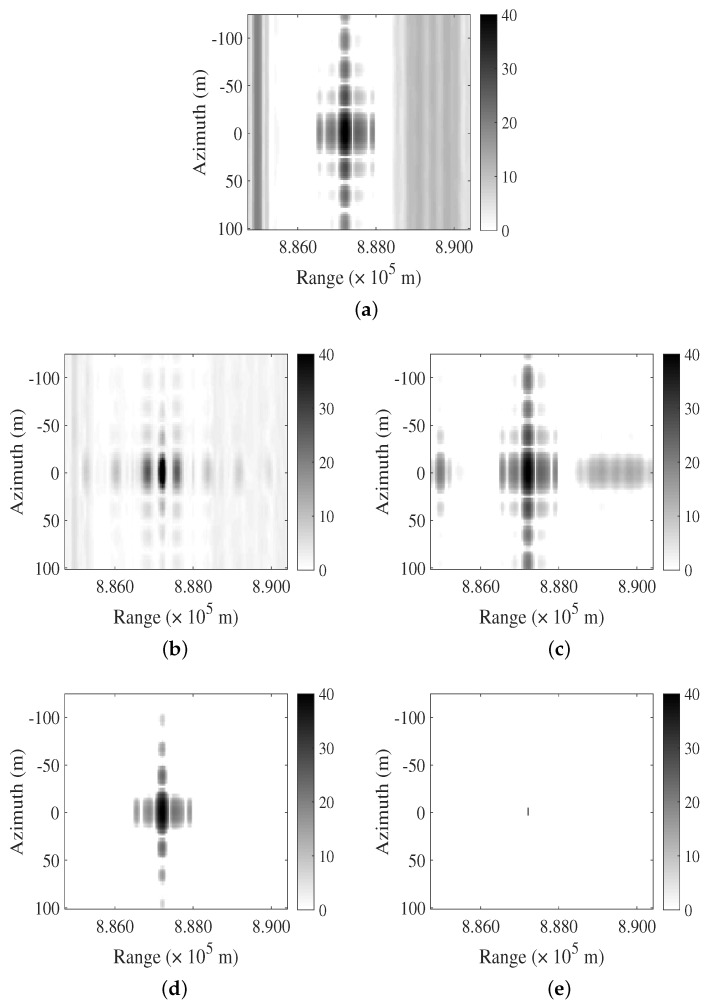
Comparison of different algorithms for suppressing LFM interference in single-point target case. (**a**) The original SAR image. (**b**–**e**) Resulting images after interference suppression using FNF, PCA, RPCA, and LFMIS-JointSR algorithms, respectively.

In the first case, a point target and two LFM interferences, namely ${\mathrm{RFI}}_{1}$ and ${\mathrm{RFI}}_{2}$, are present, as depicted in Figure 1a. The parameters of the transmitted signal, ${\mathrm{RFI}}_{1}$ and ${\mathrm{RFI}}_{2}$, are provided in Table 1. The bandwidth and chirp duration set by ${\mathrm{RFI}}_{1}$ and ${\mathrm{RFI}}_{2}$ closely align with the transmitted signal, and the signal-to-noise-plus-interference ratio (SINR) is 2.5754 dB. The noise is Gaussian white noise. The processing results of the FNF, PCA, RPCA, and LFMIS-JointSR methods are displayed in Figure 1b–e. The FNF method effectively reduces the interference energy but also diminishes the energy of the target, resulting in a reduction in the target’s amplitude. The PCA algorithm is designed to retain the main signal components to suppress interference. It does not exhibit a satisfactory interference suppression effect in the presence of ${\mathrm{RFI}}_{1}$ and ${\mathrm{RFI}}_{2}$. This is due to the similarity in parameters between the LFM interference and the transmitted signal. Both the RPCA and LFMIS-JointSR algorithms demonstrate interference suppression capabilities. Figure 1d–e reveal that the LFMIS-JointSR algorithm achieves superior interference suppression compared to other methods.

**Figure 2:**
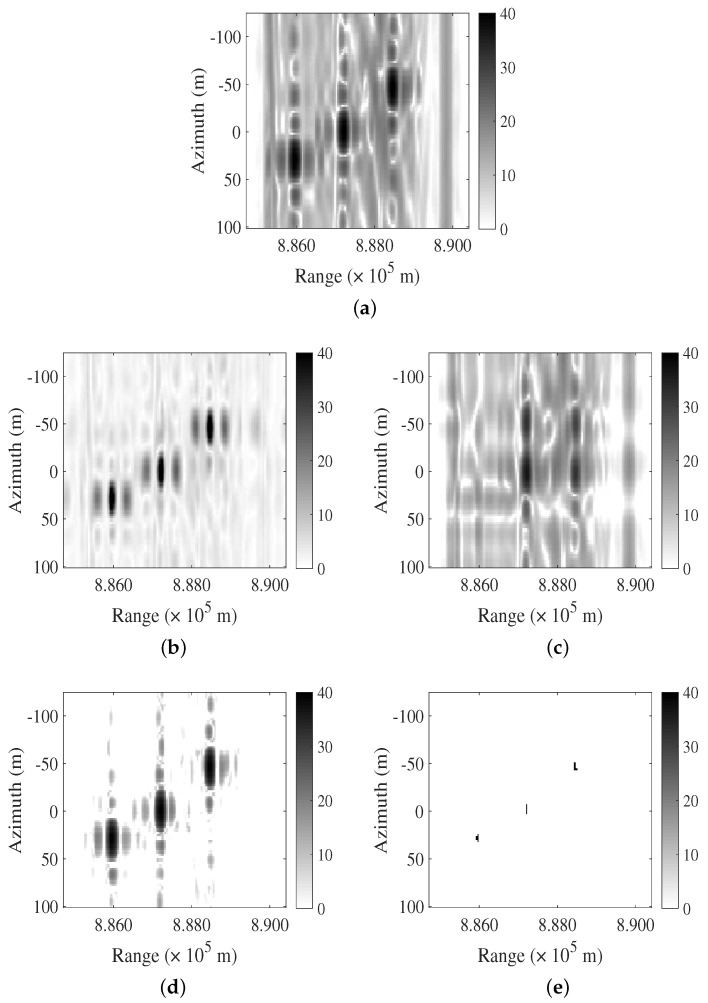
Comparison of different algorithms for suppressing LFM interference in multiple point targets and interference case. (**a**) The origrinal SAR image. (**b**–**e**) Resulting images after interference suppression using FNF, PCA, RPCA, and LFMIS-JointSR algorithms, respectively.

In the second case, three-point targets are present, as depicted in Figure 2a. Pulse LFM interferences with ${\mathrm{RFI}}_{1}$ or ${\mathrm{RFI}}_{2}$’s parameters, each consisting of three pulses, overlap with the echoes of the point targets. The SINR is 1.0234 dB. The FNF and PCA algorithms exhibit poor interference suppression effects, while the RPCA algorithm still exhibits residual interference energy. The LFMIS-JointSR algorithm consistently maintains interference suppression capabilities.

### 4.1. The Extended Target Case

**Figure 3:**
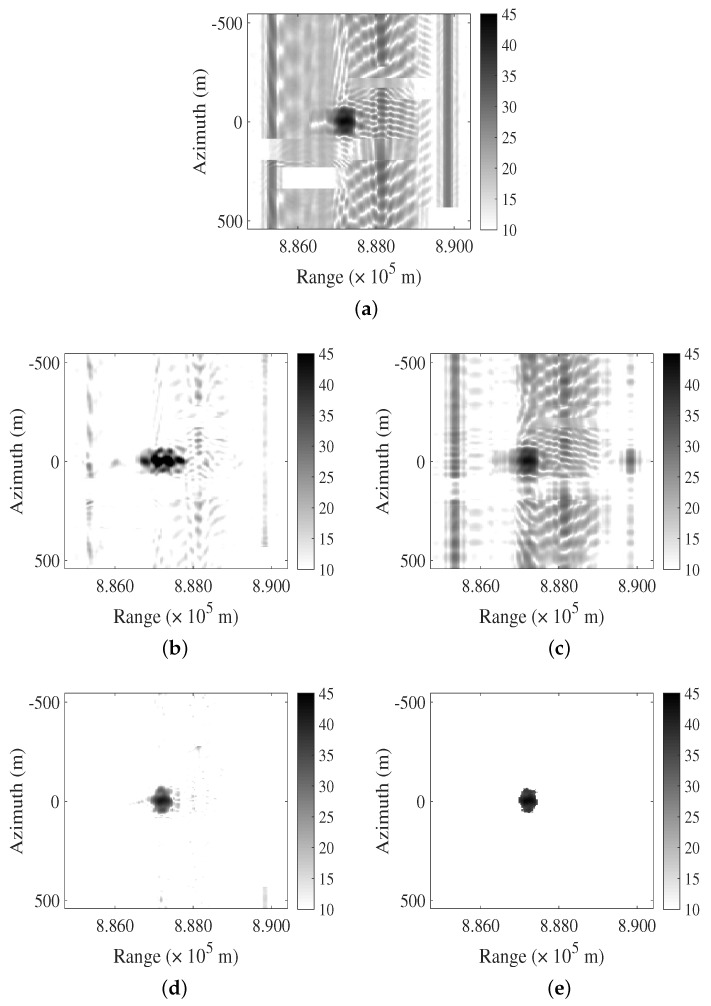
Comparison of different algorithms for suppressing LFM interference in extended target and multiple interference case. (**a**) The original SAR image. (**b**–**e**) Resulting images after interference suppression using FNF, PCA, RPCA, and LFMIS-JointSR algorithms, respectively.

The primary objective of simulation in the case of point targets is to validate the feasibility of the algorithm. In the case of interference suppression simulation with complex backgrounds, the main purpose is to evaluate the algorithm’s performance. In Figure 3a, a simulated SAR image is shown wherein the imaging of extended targets is overlapped with LFM interference. The SINR is −1.9719 dB. Figure 3b–e depict the results obtained by the FNF, PCA, RPCA, and LFMIS-JointSR methods, respectively. The results of the FNF and PCA algorithms indicate that, in the presence of complex backgrounds, neither in the frequency domain nor in the feature domain can interference and signal be effectively separated, resulting in suboptimal interference suppression.However, the RPCA and LFMIS-JointSR algorithms demonstrate the capability to suppress interference in complex backgrounds. Specifically, the LFMIS-JointSR algorithm exhibits a superior performance in separating signal and interference components accurately during interference suppression, thereby providing clearer and more reliable reconstructed images.

### 4.2. Robustness Analysis

**Figure 4:**
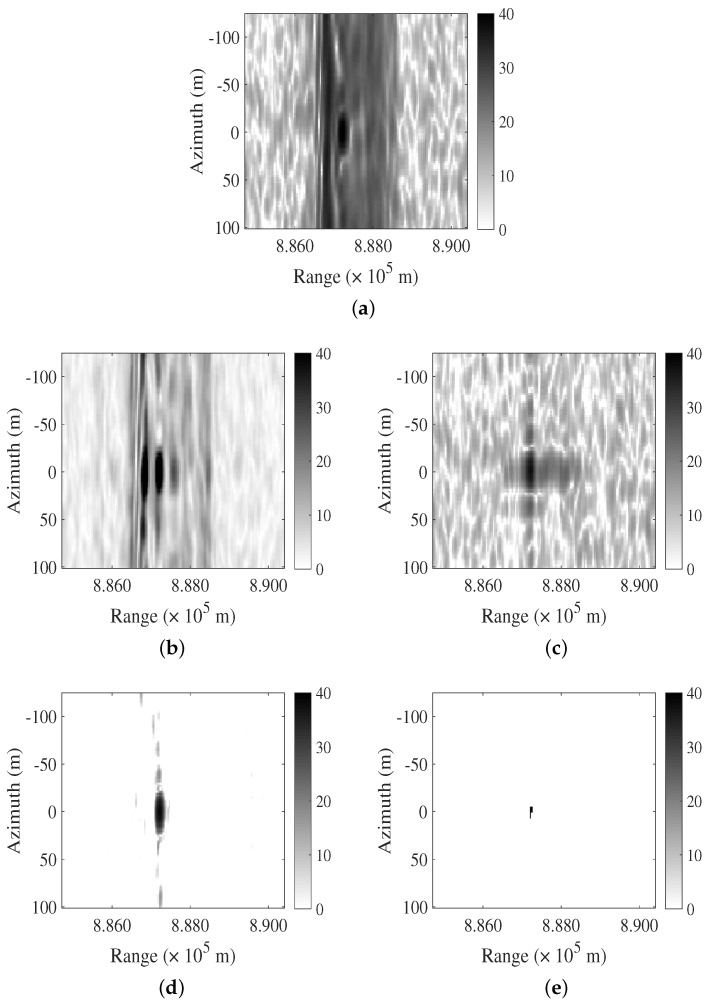
Comparison of different algorithms for suppressing LFM interference in low-SIR case. (**a**) The original SAR image. (**b**–**e**) Resulting images after interference suppression using FNF, PCA, RPCA, and LFMIS-JointSR algorithms, respectively.

The robustness of each algorithm was evaluated by comparing the resulting images after interference suppression under SIR. Figure 4a depicts a simulated SAR image with an SIR of −15 dB. In this image, there is interference present on both sides of the target point, labeled as ${\mathrm{RFI}}_{1}$ or ${\mathrm{RFI}}_{2}$, respectively, significantly impacting the observation of the target.

Figure 4b–e show the results obtained using the FNF, PCA, RPCA, and LFMIS-JointSR methods, respectively. The FNF method partially removes the interference energy in the frequency domain. The interference and target signals are not completely separated in the frequency domain, leading to a reduction in target energy while residual interference energy remains. On the other hand, the PCA, RPCA, and LFMIS-JointSR methods all demonstrate interference suppression capabilities, indicating their robustness to interference. Among them, the LFMIS-JointSR algorithm exhibits the best interference suppression performance under low SIR conditions, highlighting its strong robustness.

### 4.3. Simulation with a Real SAR Image

**Figure 5:**
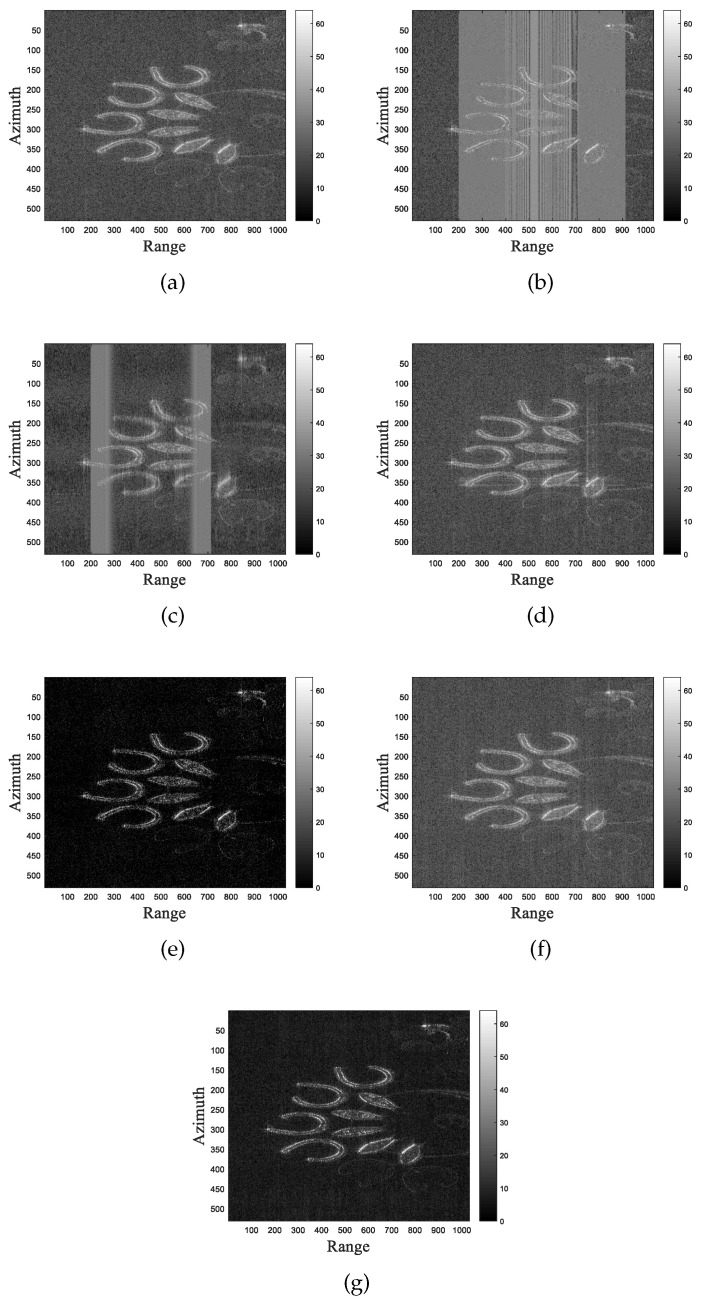
Comparison of different algorithms for suppressing LFM interference in real SAR image. (**a**) The original SAR image. (**b**) The SAR image with RFI. (**c**–**g**) Resulting images after interference suppression using FNF, PCA, RPCA, 2-D SPECAN, and LFMIS-JointSR algorithms, respectively.

In this section, we also conducted a quantitative comparison with a recently published similar work [32]. The work in [32] proposes a 2D algorithm that aims to suppress LFM interference in the SAR image domain. However, this algorithm is only effective for LFM interference with same parameters. To achieve optimal performance in this simulation, we manually executed the algorithm twice. As shown in Figure 5, the SAR image used in the experiment is the SLC image of the Sentinel-1 satellite. The image contains approximately 30 dBw of noise and numerous strong scattering points. We added ${\mathrm{RFI}}_{1}$ and ${\mathrm{RFI}}_{2}$ with a power of 40 dBw to the image. Figure 5c–g show the imaging results of FNF, PCA, RPCA, 2-D SPECAN, and LFMIS-JointSR algorithms, respectively.

Figure 5d–g show that the LFMIS-JointSR, RPCA, PCA, and 2-D SPECAN algorithms achieve good interference suppression effects, with LFMIS-JointSR and RPCA algorithms showing some noise suppression capabilities. The images’ TBRs are quantitatively compared and ranked in descending order as follows: LFMIS-JointSR achieves the highest TBR at 76.2689 dB, followed by RPCA at 73.6323 dB, PCA at 65.2427 dB, 2-D SPECAN at 65.2286 dB, and FNF at 60.3189 dB. The simulation results based on the real SAR image demonstrate that the proposed algorithm can effectively suppress multiple LFM interferences and noise in SAR image domain, and it outperforms recently published similar work.

## 5. Conclusions

In this paper, we focused on mitigating LFM interference in SLC images at the L1 level of SAR satellites. We commenced by establishing an echo signal model to concentrate the LFM interference on the SAR image. We then matrixized the process and replaced the dictionary design and sampling process of sparse signals with imaging operators. By incorporating augmented variables into the LFMIS-JointSR algorithm, we simplified the optimization problem of multiple variables into the task of finding a single variable multiple times. We provided a comprehensive description of the algorithm’s operation. Through simulations, we demonstrated that the proposed algorithm effectively focuses on various LFM interference and achieves interference suppression by concentrating and isolating the interference energy. Moreover, when compared with other interference suppression algorithms, the proposed algorithm exhibited superior interference suppression capabilities and robustness.

## Figures and Tables

**Table 1:** Simulation parameters.

Parameter	Value	Parameter	Value
Transmitted waveform	LFM signal	Carrier frequency	5.405 GHz
Signal bandwidth	1 MHz	Pulse repetition frequency	2500 KHz
Chirp duration	5 μs	Range between centre of the flight track and scene center	887.23 km
Platform velocity	7249 m s^−1^	Synthetic aperture time	0.1359 s
Theoretical range resolution	150 m	Theoretical azimuth resolution	25 m
Signal bandwidth of ${\mathrm{RFI}}_{1}$	1.7 MHz *	Chirp duration of ${\mathrm{RFI}}_{1}$	10 μs
Signal bandwidth of ${\mathrm{RFI}}_{2}$	1.7 MHz *	Chirp duration of ${\mathrm{RFI}}_{2}$	10 μs

* The frequency of ${\mathrm{RFI}}_{1}$ increases, while the frequency of ${\mathrm{RFI}}_{2}$ decreases.

**Table 2:** TBRs of the FNF, PCA, RPCA, and LFMIS-JointSR for interference suppression results in simulation experiments.

Case	Method	TBR (dB)
The first-point target case	FNF	51.9474
	PCA	51.4356
	RPCA	69.1947
	LFMIS-JointSR	154.0224
The second-point target case	FNF	43.8465
	PCA	42.0622
	RPCA	56.8874
	LFMIS-JointSR	154.4594
The extended target case	FNF	50.0983
	PCA	54.4416
	RPCA	70.6983
	LFMIS-JointSR	144.1300

## Data Availability

The real SAR image presented in the study are openly available in the Earth Science Data Systems (ESDS) Program at https://www.earthdata.nasa.gov/.
